# Scalability of self-stratifying microbial fuel cell: Towards height miniaturisation

**DOI:** 10.1016/j.bioelechem.2019.01.004

**Published:** 2019-06

**Authors:** Xavier Alexis Walter, Carlo Santoro, John Greenman, Ioannis A. Ieropoulos

**Affiliations:** Bristol BioEnergy Centre, Bristol Robotics Laboratory, T-Block, Frenchay Campus, University of the West of England (UWE), Bristol BS16 1QY, United Kingdom

**Keywords:** Microbial fuel cell, Urine treatment, Scaling, Self-stratification, Power generation

## Abstract

The scalability of bioelectrochemical systems is a key parameter for their practical implementation in the real-world. Up until now, only urine-fed self-stratifying microbial fuel cells (SSM-MFCs) have been shown to be scalable in width and length with limited power density losses. For practical reasons, the present work focuses on the scalability of SSM-MFCs in the one dimension that has not yet been investigated, namely height. Three different height conditions were considered (1 cm, 2 cm and 3 cm tall electrodes). The normalised power density of the 2 cm and 3 cm conditions were similar either during the durability test under a hydraulic retention time of ≈39 h (i.e. 15.74 ± 0.99 μW.cm^−3^) and during the polarisation experiments (i.e. 27.79 ± 0.92 μW.cm^−3^). Conversely, the 1 cm condition had lower power densities of 11.23 ± 0.07 μW.cm^−3^ and 17.73 ± 3.94 μW.cm^−3^ both during the durability test and the polarisation experiment, respectively. These results confirm that SSM-MFCs can be scaled in all 3 dimensions with minimal power density losses, with a minimum height threshold for the electrode comprised between 1 cm and 2 cm.

## Highlights

• Membraneless microbial fuel cells (SSM-MFCs) with different heights were investigated.
• SSM-MFCs can be scaled-up in height without power density losses.
• SSM-MFCs cannot be scaled-down below 1 cm tall electrodes without losses.
• Stable operation under potentiostatic conditions achieved for 48 days.

## 1 Introduction

Amongst bioelectrochemical systems, the field of microbial fuel cells (MFCs) is a widely-studied topic. MFCs are of interest because they combine the production of low levels of electricity and the treatment of different types of wastewater: the reduced organic matter contained and dissolved in the waste is converted directly into electricity through the metabolic activity of anaerobic electro-active microorganisms [1,2], with the anode acting as the terminal electron acceptor. During this process, protons, smaller organic molecules and CO_2_ are released into the electrolyte. Whilst the electrons travel toward the cathode through an external load, protons, and other ions depending on the pH, diffuse toward the cathode. At the cathode, the (cat)ions and electrons react through a reduction reaction with an oxidant of a higher redox potential (e.g. oxygen) [3], to produce current (electron flow). The main advantage of this technology is that it can treat waste streams of various sources (e.g. activated sludge [4], neat urine [5] and others [6,7]), with comparable removal rates to the industry sector of wastewater management [8].

Although oxygen is the preferred cathodic oxidant because of its availability, the oxygen reduction reaction (ORR) occurring on the cathodes of bioelectrochemical systems is often the limiting reaction because of its sluggish kinetics [9,10]. This is due to the low concentration of H^+^ and OH^−^, reactants of the ORR, at neutral (or circumneutral) pH [[11], [12], [13]]. In order to enhance ORR in microbial fuel cells, a catalyst is usually used [[14], [15], [16]]. Three main types of catalyst are used: (i) platinum group metals (PGM), (ii) platinum group metal-free (PGM-free) and (iii) carbonaceous based [[14], [15], [16]]. Due to the low durability and high cost, platinum-based materials are less common [17,18]. Although PGM-free catalysts based on transition metals such as Fe, Mn, Co and Ni [19,20], have high performance and durability in long run experiments, their production cost still remains high [21]. Carbonaceous-based catalysts, on the other hand, are preferred to PGM and PGM-free and well utilized for MFCs cathodes [16,22,23], mainly due to their lower cost and despite their lower electrochemical performance. Among these carbonaceous-based catalysts, the most commonly used is activated carbon (AC), which has superior performance in neutral media than carbon black [24] and has high durability in long-term operation [25,26]. Furthermore, AC mixed with PTFE (polytetrafluoroethylene) and pasted on a stainless steel mesh is, at the current stage, the catalyst that presents (i) good performance to cost ratio, (ii) simple production process and (iii) good stability over time (i.e. mechanical and chemical resistance) [23,27].

With regard to implementation, usable power levels and enhanced treatment are obtained when a plurality of MFCs are assembled in stacks [28,29]. Developing MFC stacks requires an equilibrium between unit size, design simplicity and cost, and the recently developed concept of self-stratifying membraneless MFC (SSM-MFC) [[30], [31], [32]] seems to show such properties. The principle behind SSM-MFCs is to exploit the capacity of microorganisms to structure layers into a wet environment, each characterised by specific bio-chemical conditions (i.e. redox state of chemical elements, type of dominating metabolic activity) [33]. Here, the SSM-MFCs exploit the same phenomenon in a urine column with a plurality of cathodes being placed in the upper oxic layers and a plurality of anodes placed in the lower anoxic layers. In addition to the aforementioned advantages of this design, one of the main characteristics of these MFCs is that each unit can be scaled in width and length with negligible power density losses [30,34]. Such scalability properties offer the possibility to scale a SSM-MFC stack for a specific application whilst maintaining performance.

The concept of SSM-MFC was primarily designed to treat a particular fuel that was neat undiluted urine. In developed urban settings, urine is the source of 75%, 50% and 10% of the nitrogen, phosphorous and COD present in domestic wastewaters, respectively, whilst it only accounts for less than 5% of the total volume [35,36]. The treatment, or pre-treatment, of this type of waste stream prior to reaching wastewater treatment plants is therefore an attractive solution [36,37] that could perhaps reduce the energy consumption of the wastewater treatment process [5,30,[38], [39], [40]]. Besides urea ((NH_2_)_2_CO), which accounts for 50% of the organic carbon contained in urine, up to 3079 different metabolites are also present [41]. When urine is fed to dual compartment MFCs, these organic compounds are degraded in the anode [42]. During the first 24 h, urea is primarily hydrolysed by urease enzymes into ammonia (NH_3_) and carbamate (NH_2_COOH). The carbamates are then further degraded into ammonia and carbon dioxide [43]. To the authors' best knowledge, the MFC is the only biotechnology able to directly treat neat urine – with no dilution at any step of the process, and without inhibition due to high nitrogen concentrations – which also does not require any energy input [34].

A cascade is defined as a set of modules where the output of one unit is feeding into the input of the next one. As shown by previous studies, the more units a cascade comprises, the more efficient is the treatment [28,29]. Consequently, any implementation would benefit from having shallower units. However, all the studies on SSM-MFCs employed units with similar height (i.e. ≈10 cm urine column depth) [27,[30], [31], [32],^34^]. Hence, the impact of downscaling the height of such unit is yet unknown. Hence, the aim of the present study was to evaluate the height scalability of SSM-MFCs using 3 heights in duplicate. As the cathode and anode electrodes had the same height and as they were always at the same distance from each other (i.e. 5 mm), the experimental conditions were labelled by the height of a single electrode: 1 cm, 2 cm and 3 cm. Similar to the previous studies, the cathodes were based on a carbonaceous-based catalyst pressed over stainless steel (grade 316) current collectors. Because of its relatively low cost and high electrocatalytic activity toward oxygen reduction reaction (ORR) in neutral media, activated carbon was chosen as the catalyst and PTFE as the binder because of the ease of production, low cost and high durability [27]; the anodes were made with 10 g·m^−2^ carbon veil, as previously reported. Once inoculated, the SSM-MFCs were maintained under continuous flow of urine and at a constant potential of 450 mV. Then the electrochemical properties of the SSM-MFCs were investigated. Electrocatalytic activities of the cathodes were initially assessed in “clean” media and then tested also in operating SSM-MFCs. Polarisation and power curves were recorded after steady states were reached and also following an additional 30 days, in order to study longevity.

## 2 Material and methods

### 2.1 Reactors construction and operation

**Scheme 1:**
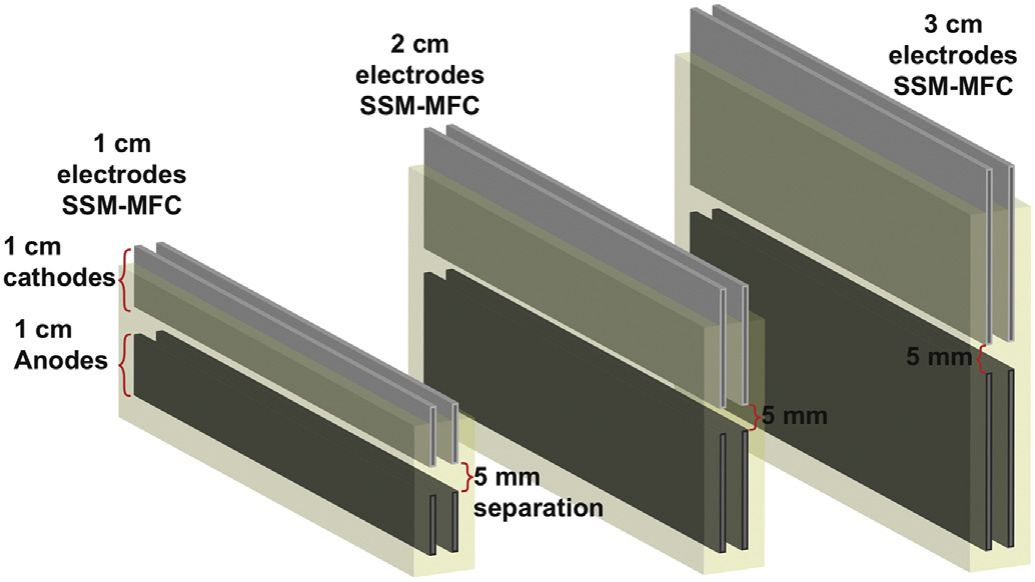
Representation of the 3 tested conditions: SSM-MFC with 1 cm, 2 cm and 3 cm tall electrodes. Both cathodes and anodes are submerged in the same electrolyte (i.e. urine represented in yellow).

Each SSM-MFC employed in this work comprised 2 cathodes placed above 2 anodes. Both anodes and both cathodes were connected in parallel. The structure of the SSM-MFCs was similar to previous laboratory studies [27,32]. Three height conditions were tested in duplicates and all error bars indicated in the manuscript stand for the range of these duplicates: the duplicate SSM-MFCs were mounted with electrodes of 1 cm, 2 cm or 3 cm (see Scheme 1.) The space between anodes and cathodes was kept at 5 mm for all three tested heights. All electrodes were 6 cm in length. The cathodes consisted of a AC-PTFE (activated carbon (AC); polytetrafluoroethylene (PTFE)) mixture pressed on a stainless-steel 316 mesh (8 × 8 mesh; MeshDirect, UK) as previously described [[30], [31], [32],^34^]. The final weight of the single cathodes was of 2.34 ± 0.05 g for the 1 cm SSM-MFCs with a total surface area of 24 cm^2^ (2 cathodes with 6 cm^2^ per side), 5.64 ± 0.16 g for the 2 cm SSM-MFCs with a total surface area of 48 cm^2^ (2 cathodes with 12 cm^2^ per side), and 8.64 ± 0.21 g for the 3 cm SSM-MFCs with a total surface area of 72 cm^2^ (2 cathodes with 18 cm^2^ per side). The AC/PTFE loading on the cathodes was 186 ± 7 mg.cm^−2^ (80% AC; 20% PTFE). Each anode was fabricated using carbon fibre veil (10 g.m^−2^; Technical Fibre Products Ltd., Cumbria, UK): the surface area of carbon veil employed for a single anode was 180 cm^2^ (6 cm × 30 cm) for the 1 cm high anodes, 354 cm^2^ (6 cm × 59 cm) for the 2 cm high anodes, and 528 cm^2^ (6 cm × 88 cm) for the 3 cm high anodes. The anodes of the 1 cm, 2 cm and 3 cm electrodes were folded down to a projected surface area of 6 cm^2^ (6 cm × 1 cm), 12 cm^2^ (6 cm × 2 cm), and 18 cm^2^ (6 cm × 3 cm), respectively. A strip of stainless steel 316 mesh (6 cm × 1 cm) was fixed in the middle of each anode to act as both a current collector and a structural support.

Because the height of the electrodes was different for the three tested conditions, the displacement volume of urine in the tested SSM-MFCs was also different: the 1 cm SSM-MFCs had a volume of 47 ± 2 ml; the 2 cm SSM-MFCs had a volume of 69 ± 3 ml; the 3 cm SSM-MFCs had a volume of 89 ± 1 ml. In terms of scale-up, the ratios of MFC volume correspond to 1.5 (1 cm vs 2 cm) and 1.9 (1 cm vs 3 cm), thus illustrating the need for normalisation. Moreover, because the SSM-MFCs were continuously fuelled by the same multichannel peristaltic pump (Watson & Marlow LTD, UK), the hydraulic retention time of each tested condition (HRT) was set to equivalent levels, by employing manifold tubing of different internal diameter. Due to these pumping tubes being from commercially available sizes, there was some variation between the HRT of the tested depths: the 1 cm SSM-MFCs had a HRT of ≈2323 min (≈38.72 h); the 2 cm SSM-MFCs had a HRT of ≈2324 min (≈38.73 h); and the 3 cm SSM-MFCs had a HRT of ≈2346 min (≈39.10 h). As indicated by the results, these differences were not correlated to the performance of the SSM-MFCs. During the initial phase of inoculation, resistive loads were applied in proportion to the amount of electrode for each tested condition: a 1125 Ω load was applied to the SSM-MFCs having 1 cm tall electrodes, a 562 Ω load was applied to the SSM-MFCs having 2 cm tall electrodes and a 375 Ω load was applied to the SSM-MFCs having 3 cm tall electrodes for roughly 5 days. Once the units reached steady state, they were connected to an electronic controller that maintained a constant potential of 450 mV (see § 2.2).

All the electrodes were submerged in the same undiluted human urine electrolyte. The urine level was adjusted to approximatively ¾ of the cathode height, as shown previously to be the best performing condition [32]. However, the bioreactors displayed some variation in HRT, which was not strictly at 1×, 2× and 3× for the three sizes, respectively. Once the SSM-MFCs were built and under constant running conditions, the cathode surface area exposed to air was measured and the final cathode wet surface area calculated for each tested condition: the SSM-MFCs having 1 cm electrodes had a cathode wet surface area of ≈19.2 cm^2^, the SSM-MFCs with 2 cm electrode had a cathode wet surface area of ≈28.8 cm^2^, and the 3 cm bioreactors had a cathode wet surface area of ≈48.0 cm^2^. In terms of scale-up, the ratios of the cathode wet surface area correspond to 1.5 (1 cm vs 2 cm) and 2.5 (1 cm vs 3 cm), thus illustrating the need for normalisation. To avoid confusion, the tested conditions will be referred to as condition A, B and C as described in Table 1.

**Table 1:** Parameters of the SSM-MFCs under the three tested conditions.

Conditions	Displacement volume	Volumetric scaling ratio	Electrodes heights	Cathode wet surface area	Cathode wet surface area scaling ratio	Hydraulic retention time
A	47 ± 2 ml	1	1 cm	≈19.2 cm^2^	1	≈2323 min
B	69 ± 3 ml	1.5	2 cm	≈28.8 cm^2^	1.5	≈2324 min
C	89 ± 1 ml	1.9	3 cm	≈48.0 cm^2^	2.5	≈2346 min

Urine was collected daily from a tank pooling together the excreta donated by anonymous and healthy individuals with no known previous medical conditions. As the urine was kept in a tank (max. 24 h), it had already undergone partial hydrolysis by the naturally occurring microflora. Due to this partial hydrolysis, the pH and electrolyte solution conductivity (EC) of the urine had values ranging between 8.5 and 9.3 and 28 ± 2 mScm^−1^, respectively. Human urine generally comprises 4.7–10.4 g·L^−1^ dry matter of which 65–85% are organic compounds; urea being the main constituent of the total organic solids (≈50%) [44]. The SSM-MFCs were inoculated with a mixture consisting of 50% (*v*/v) of the effluent of a separate, mature MFC [32], also fed with urine, and 50% (v/v) of freshly collected urine.

### 2.2 Data capture and system characterisation

The fuel cells in this experiment had a fixed external Constant Voltage Load attached. The constant voltage applied was 450 mV, and a mechanism using an operational amplifier (TSZ124IQ4T) with a reference voltage would load the MFC through an N-channel MOSFET (BSS138) in order to sink current. This custom electronics unit would not load the MFC if the voltage dropped below 450 mV, and would dynamically adjust the load with increasing MFC output levels. Due to the internal resistance of the MFC, the increased resistive load on the output allows the voltage to be kept constant. With the addition of a series shunt resistor and current amplifier, the load applied by the constant voltage load can be measured by the Agilent Data Acquisition System (Agilent LXI 34972A; Farnell, UK), and logged over time every 4 min. Since the acquired voltage was an indirect measurement of the current, the following formula was used to calculate the measured current:

(1) $$I=\frac{\left(V_{m}-1.20\right)}{19.8}$$

Where *I* is the current in Amperes (A) and *V*_*m*_ is the voltage measured in Volts (V) by the acquisition system (i.e. Agilent 34972A). The power *P* in Watts (W) produced by each unit was calculated using the formula, *P = I x V*, where *V* is the constant voltage (450 mV) in Volts (V) and *I* is the calculated current using Eq. (1).

To compare the SSM-MFCs and verify the hypothesis of scalability, the current and power values were normalised either by the whole electrolyte displacement volume or by the cathode wet surface area. The volumes, by which the data were normalised, were 47 ± 2 ml, 69 ± 3 ml and 89 ± 1 ml for the 1 cm, 2 cm and 3 cm conditions, respectively. The cathode wet surface areas used for the 1 cm, 2 cm and 3 cm conditions were ≈19.2 cm^2^, ≈28.8 cm^2^ and ≈48.0 cm^2^, respectively. These wet surface areas were measured once the SSM-MFCs were set and put under continuous flow, as described in the previous section.

The initial polarisation of the cathodes was performed in phosphate buffer (pH=7.06; EC = 14.86 mS·cm^−1^) after being in contact with the electrolyte for over 24 h in order to avoid the presence of oxygen adsorbed on the activated carbon surface. The polarisation runs were performed running a linear sweep voltammetry (LSV) using a Biologic potentiostat (SP-50, France) under a three-electrodes configuration with the anodes being used as counter electrodes, the cathodes as the working electrodes and the Ag/AgCl (3 M KCl) as the reference electrode being placed next to the cathode to reduce the ohmic resistance given by the electrolyte. Since MFC are complex electrochemical system, the scan rate was slow (0.25 mV·s^−1^) in order to avoid overestimation of the performance [45]. The LSVs were run between open circuit potential (OCP) to −400 mV.

SSM-MFC was firstly disconnected from the constant load device for at least 1 h. After the open circuit voltage (OCV) was stabilised, polarisation curves of the mature SSM-MFC was performed running LSVs in a two-electrode configuration (potentiostat Biologic SP-50) whereby the reference electrode channel was short-circuited with the counter electrode channel, the anode was the working electrode and the cathode served as the counter electrode. The scan rate was 0.25 mV·s^−1^ and ranged from OCV to 50 mV. Polarisation curves were also run for each single electrode (anode and cathode) using a three electrodes configuration. Once again, before each polarisation, SSM-MFC were left in OCV for at least 1 h. The anode polarisation curves were measured using the anode as the working electrode, cathode as the counter electrode and Ag/AgCl (3 M KCl) as the reference electrode. Anodic LSVs were run between anode OCP and −100 mV (vs Ag/AgCl). The cathode polarisation curves were run using the cathode as working electrode, anode as counter electrode and Ag/AgCl (3 M KCl) as reference electrode. Cathodic LSVs were run between cathode OCP and −200 mV (vs Ag/AgCl). During these polarisation curves, the reference electrode was placed closed to the working electrode in order to minimise the effect of the electrolyte ohmic resistance on the current measured. All the LSVs were run at a scan rate of 0.25 mV·s^−1^.

## 3 Results and discussion

### 3.1 Cathode polarisation curves in clean media

The cathodes were initially characterised in clean media (i.e. phosphate buffer solution, pH = 7.06; EC = 14.86 mS·cm^−1^) through a polarisation curve ran in a three-electrode configuration as described in Section 2.2. Concerning the scalability of the electrodes, the hypothesis being that because the proportion of cathode surface area in contact with the liquid is the same for all tested conditions, these then should display similar current density. Since the urine column heights are proportional to the size of the tested bioreactors, this implies that the cathode surface area in contact with the liquid electrolyte is proportionally the same, independent of the height of the bioreactors, and therefore the oxygen reduction reaction (ORR) rate should be similar.

**Fig. 1:**
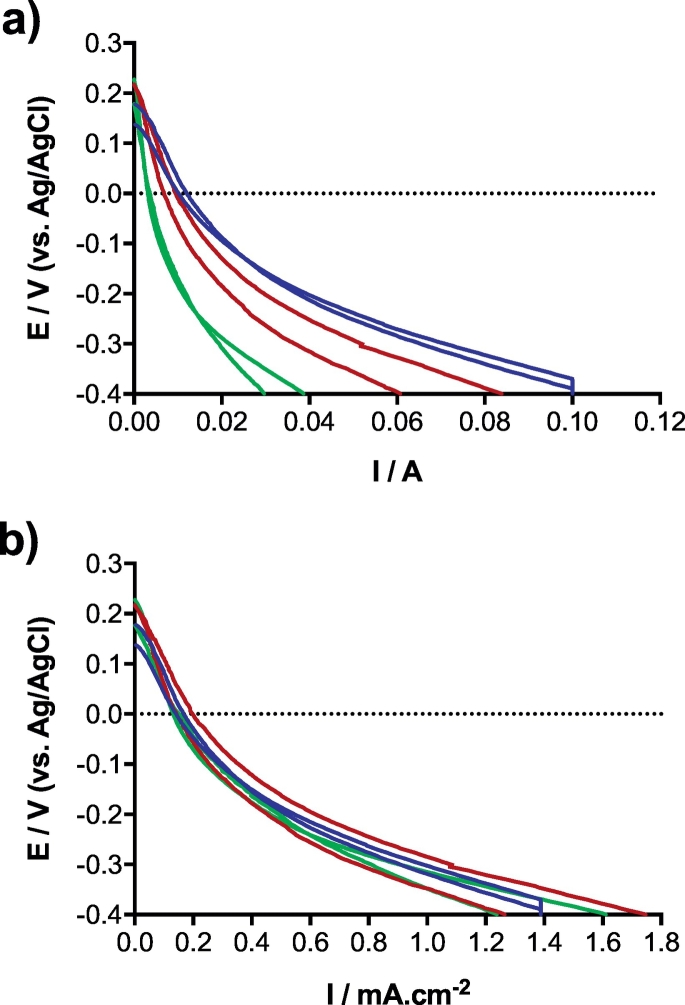
Polarisation curves of the cathodes in phosphate buffer (pH = 7.06; EC = 14.86 mS·cm^−1^) prior to inoculation. Lines represent duplicate of independent reactors. Green, red and blue curves stand for the 1 cm, 2 cm and 3 cm conditions, respectively conditions A, B and C. (For interpretation of the references to colour in this figure legend, the reader is referred to the web version of this article.)

Fig. 1 shows that the initial open circuit potentials (OCP) of the polarisation curves were similar and started at 202 ± 45 mV vs Ag/AgCl. This was expected since the OCP is a thermodynamic characteristic that is independent from the size of electrodes. Considering that the theoretical potential value of the ORR at neutral pH (equal to 7) is roughly +600 mV vs Ag/AgCl (3 M KCl), the activation overpotentials of these cathodes were ≈400 mV. These results are similar or slightly lower than previously reported work, utilising activated carbon based cathodes [32,46,47]. It was expected that the larger the cathode surface area exposed to the liquid, the greater the number of catalytic sites in contact with the electrolyte and therefore higher levels of current should be produced. Since the cathode potential never reached negative values in a working microbial fuel cell, the comparison of the polarisation curves is made at 0 mV potential. The results from these polarisation runs show an increase in electrocatalytic activity when the height of SSM-MFC increases from 1 cm, to 3 cm (Fig. 1a). In particular, the current produced at 0 mV (vs Ag/AgCl), for the 1 cm cathode was 3.37 ± 0.21 mA, for the 2 cm was 8.14 ± 1.28 mA and for the 3 cm 11.01 ± 0.92 mA. This corresponds to an enhancement of 142% and 226%, for the 2 cm and 3 cm, respectively. These linear results are expected since the active surface of cathode (i.e. surface in contact with the electrolyte) is larger in taller SSM-MFCs, and so is the ORR.

However, as the work focuses on the scalability of the system, it is the current density that will confirm or not the hypothesis. To verify if the system is scalable in height, the current was normalised by the cathode wet surface area. As there were two cathodes per SSM-MFC and the level of urine was adjusted to ≈¾ of the cathode height, the cathode wet surface areas for the 1 cm, 2 cm and 3 cm cathodes were ≈19.2 cm^2^, ≈28.8 cm^2^, and ≈48.0 cm^2^, respectively. Results show that the differences of current density measured during the cathode polarisation experiments were not significant (Fig. 1b). Particularly, considering the current density at 0 mV vs Ag/AgCl, all the tested conditions had an average current density of 0.15 ± 0.02 mA.cm^−2^. At that stage and in clean media, results indicate that the SSM-MFCs are scalable in height without current density losses (Fig. 1b). Results of these polarisation curves show that in clean media the cathode open circuit potential (OCP) was independent from the bioreactor configuration.

### 3.2 Temporal power production under potentiostatic conditions

**Fig. 2:**
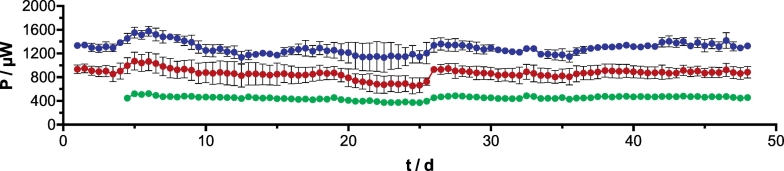
Temporal absolute power evolution of the SSM-MFCs under potentiostatic conditions (450 mV). Although data points were recorded every 4 mins, for clarity purposes, only two values per day are plotted. The curves are the average of each duplicate condition and the error bars stand for the values range. Green, red and blue curves stand for the 1 cm, 2 cm and 3 cm conditions, respectively conditions A, B and C. (For interpretation of the references to colour in this figure legend, the reader is referred to the web version of this article.)

**Fig. 3:**
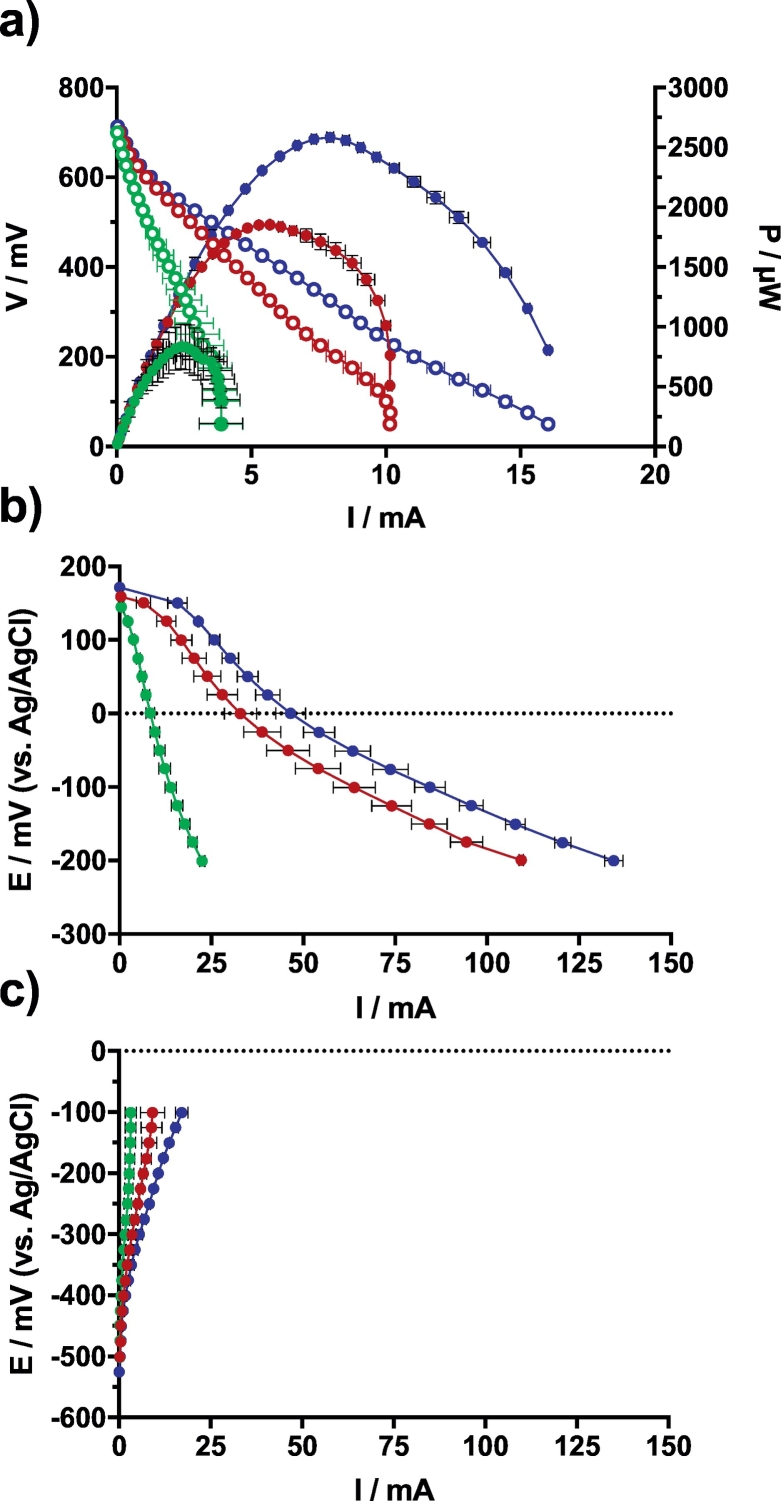
Electrochemical characterisation of the mature SSM-MFCs. a) Overall polarisation of the SSM-MFCs from OCV. The Voltage-current polarisation curves are shown by empty circles whilst the power-current curves are illustrated by plain circles. b) Potential-current polarisation curves of the cathodes. c) Potential-current polarisation curves of the anodes. The curves are the average of each condition duplicates and the error bars stand for the values range. Green, red and blue curves stand for the 1 cm, 2 cm and 3 cm conditions, respectively conditions A, B and C. (For interpretation of the references to colour in this figure legend, the reader is referred to the web version of this article.)

Following the cathode polarisation curves, the SSM-MFCs were inoculated using the effluent of other mature MFCs also fed with urine. The test MFCs were then left in open circuit for 5 h prior to connecting the load in order to allow the stratification of the system. Such stratification allows the creation of two liquid strata, the bottom one where the anodes are being reduced, and the top layer of the column, where the cathodes are oxidised. As the SSM-MFCs were placed under continuous flow of fuel, once the produced power plateaued, it was considered that the SSM-MFCs reached steady state. Once the steady states were reached, the SSM-MFCs were placed under potentiostatic conditions (450 mV) for 48 days (Fig. 3). Results from the temporal experimental run show that the taller the electrodes are, higher aboslute power is produced. The SSM-MFCs with A conditions produced, at day 40, 528 ± 3 μW, the SSM-MFCs with B conditions produced 1038 ± 46 μW, and the SSM-MFCs with C conditions produced 1480 ± 17 μW (Fig. 2). Compared to the SSM-MFC under A conditions, doubling the electrode height resulted in a power increase of 96% whilst tripling the electrode height resulted in 180% power increase. Interestingly, this power increase is approximately linearly proportional to the amount of electrode contained by the SSM-MFCs: doubling the electrode size resulted in nearly double the overall power. However, since the SSM-MFCs were not precisely scaled up, they displayed some variations in electrolyte volume, thus in wet cathode surface area, which has a direct impact on the performance [32]. Hence, to confirm the scalability of SSM-MFCs, the electrochemical performance need to be normalised by both the volume of electrolyte and by the cathodes wet surface area (i.e. electroactive part of the cathodes; Table 1).

### 3.3 Electrochemical characterisation of the matured SSM-MFCs

After a steady state of 48 days (Fig. 2), the SSM-MFCs were characterised through a linear sweep voltammetry experiment ran in a two-electrode configuration, whilst the electrodes were characterised through a polarisation experiment ran in a three-electrode configuration. The polarisation experiments were run after leaving the SSM-MFCs during 1 h under open circuit conditions. The anode and cathode potentials were recorded separately (2 different polarisation experiments) against a Ag/AgCl (3 M KCl) reference electrode placed either next to the cathode or next to the anode in order to minimise the ohmic losses of the electrolyte [48]. During the cathode polarisation, the anode served as the counter electrode and vice versa during the anode polarisation, the cathode served as the counter electrode.

The SSM-MFCs were operating with the electrolyte having a constant pH of ≈9.3. The theoretical open circuit voltage (OCV) of an operating MFC is ≈1120 mV. This is the consequence of the redox potential difference between the cathodic reaction involving the oxygen (≈ + 470 mV (vs Ag/AgCl) at pH = 9.3) and the anodic organic oxidation reaction performed by the anaerobic microbiota, assuming that only the NADH is involved (≈−650 mV vs Ag/AgCl at pH = 9.3). As shown by the polarisation curves, the SSM-MFCs displayed similar OCV levels (Fig. 3a), a parameter that is independent from the size of the electrodes. The A conditions had an OCV value of 693 ± 1 mV, whilst the B and C conditions had OCV levels of 707 ± 3 mV and 715 ± 3 mV, respectively. Compared to the theoretical value, the SSM-MFCs had 40% lower OCV levels. Nonetheless, these values are in agreement with previously published results [24,32].

From an implementation point of view, the maximum power produced by the MFCs is a key parameter. The polarisation curves shown in Fig. 3a demonstrate that larger bioreactors had a higher maximum power than the smaller one. In comparison to the A conditions that produced a maximum of 834 ± 191 μW, the B and C conditions produced 1854 ± 8 μW and 2584 ± 39 μW, respectively. This corresponds to 123% more power from the SSM-MFCs under B conditions and 210% from the C conditions, which is in agreement with the size increase. However, it is worth noting that only the A conditions demonstrate large variations in power output when compared to the B and C conditions (i.e. ±191 μW, ±8 μW, 39 μW respectively). This is due to the fact that within the duplicate of the A conditions, one MFC was underperforming (643 μW) and the other was performing similarly to the other conditions by proportionate comparison of the wet cathode surface area (1025 μW). A larger variation in the output compared with the other conditions, and taking into account the variability in the build at such a small scale, suggest that SSM-MFC mounted with 1 cm electrodes is the limit at which stratification does not fully occur.

The results from the cathode polarisation experiment indicate that the A, B and C conditions displayed increasing open circuit potential (OCP) that was 147 ± 5 mV vs Ag/AgCl, 159 ± 2 mV vs Ag/AgCl, and 186 ± 9 mV vs Ag/AgCl, respectively. These are typical values, as previously published [24,32]. This light difference might be explained by the fact that biofilm was growing on the cathode electrodes decreasing the electrode surface accessibility to available oxygen. As expected from electrodes with higher wet surface area (i.e. part of the cathode in contact with the electrolyte), the cathode polarisation curves of the larger SSM-MFCs are higher than that of the smaller one (Fig. 3b). However, the cathode curves are not proportional to the electrode height increase. Compared to the A conditions that produces 8.4 ± 1.2 mA at 0 mV (vs Ag/AgCl), the B and C conditions produce 32.9 ± 4.4 mA and 46.6 ± 4.0 mA at 0 mV (vs Ag/AgCl), respectively. These results correspond to a 292% and 455% current increase, respectively.

The anode OCP of the tested conditions are somewhat similar but are increasingly negative with the size of the bioreactors. The anode OCP of the A, B and C conditions are of −483 ± 16 mV vs Ag/AgCl, −512 ± 15 mV vs Ag/AgCl and −535 ± 7 mV vs Ag/AgCl, respectively. These higher anode OCPs could suggest that oxygen or other oxidants (e.g. nitrate) could interfere with the anode functioning, therefore explaining the higher OCP of shallower SSM-MFCs. This trend was also suggested previously, where the OCP increased due to lower distance from the electrode to the exposed air meaning less column of urine above the anode [11]. The anode polarisation curves shown in Fig. 3c indicate that, as for the power curves and the cathode polarisation curves, the SSM-MFCs with larger surface area of anode produce more current. However, the most important result is that in the tested SSM-MFCs the anodes are limiting the system performance. At a potential of −100 mV (vs Ag/AgCl), the anodes of the A conditions produced 22% of the current produced by the cathodes (3.18 ± 1.57 mA vs 13.93 ± 1.56 mA) (Fig. 3b,c). Similarly, the anodes of the B conditions only produced 14% of the current produced by cathode (i.e. 9.16 ± 3.31 mA vs 63.85 ± 5.75 mA) and the anodes of the C conditions only produced 20% of the current compared to the cathode (i.e. 17.11 ± 1.68 mA vs 84.45 ± 4.17 mA) (Fig. 3b,c). These results indicate that to increase the system performance independently from the size of the reactor, more surface area of carbon veil anode should be added for the same surface area of cathode employed.

### 3.4 Normalisation of the electrochemical polarisation results

**Fig. 4:**
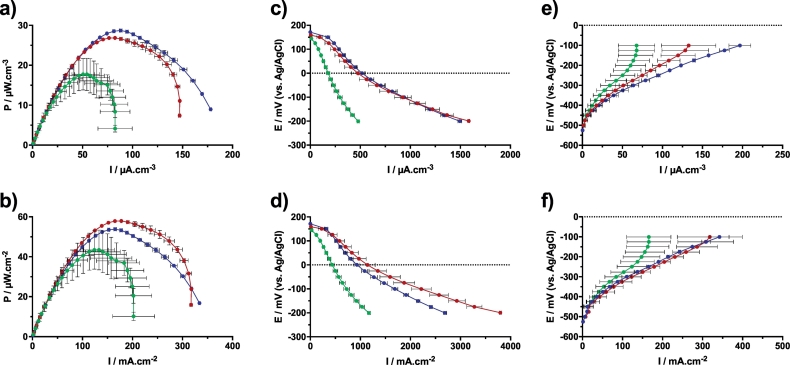
Normalised data of the electrochemical characterisation results. Power curves of the matured SSM-MFCs normalised by the total volume of electrolyte (a) and by the cathode wet surface area (b). Normalisation of cathode polarisation curves by the total volume of electrolyte (c) and by the cathode wet surface area (d). Normalisation of anode polarisation curves by the total volume of electrolyte (e) and by the cathode wet surface area (f). Green, red and blue curves stand for the 1 cm, 2 cm and 3 cm conditions, respectively conditions A, B and C. (For interpretation of the references to colour in this figure legend, the reader is referred to the web version of this article.)

In order to verify the hypothesis that SSM-MFCs can be scaled in height without decreasing the electrochemical performance, the results from the polarisation experiments were normalised. Such normalisation allows to homogenise the results independently from the physical constrains of the small bioreactors employed in this study (e.g. urine levels, volume of electrolyte). Therefore, the polarisation results were normalised by the measured volumes and cathode wet surface areas (Fig. 4).

The volumetric normalisation was performed using the displacement volume of electrolyte, which was 47 ml, 69 ml and 89 ml for the A, B and C conditions, respectively (Table 1). The cathode surface area normalisation was performed using the cathode wet surface area that was 19.2 cm^2^, 28.8 cm^2^ and 48.0 cm^2^, for the A B and C conditions, respectively.

The normalisation power curves, either by volume (Fig. 4a) or by cathode wet surface area (Fig. 4b), show similar results. The B and C conditions display similar power density whereas the A conditions has a much lower power density (Fig. 4a,b). At the maximum power transfer point the B and C conditions had power densities of 27.79 ± 0.92 μW.cm^−3^ and 55.89 ± 2.06 μW.cm^−2^. Conversely, the A conditions had a maximum power density of 17.73 ± 3.94 μW.cm^−3^ and 43.41 ± 9.64 μW.cm^−2^. As mentioned above for the A conditions, there is a large variation in the output levels. This might be due to the 1 cm electrode height being the potential limit at which the redox potential stratification is not well defined, and therefore not in accordance to the physical structure of the system. Interestingly, the volumetric power density of the SSM-MFCs mounted with 3 cm tall electrodes were slightly higher than that of the B conditions (Fig. 4a), whereas the normalisation by the cathode wet surface area shows that the SSM-MFC mounted with 2 cm electrodes had slightly higher power density than the C conditions (Fig. 4b). This observation can be explained by the electrochemical behaviour of the single electrodes. The higher volumetric power density of the C conditions was due to a better performance of the anode (Fig. 4e) since the cathode polarisation curves were similar (Fig. 4c). Conversely the higher power density of the B conditions, when normalised by the cathode wet surface area, was due to better cathode electrochemical behaviour of the cathode (Fig. 4d) since the anode curves were similar (Fig. 4f).

Independently from the normalisation parameter used, the A conditions always had lower electrochemical performance than the B and C conditions (Fig. 4). Since, under the tested running conditions, the anodes were the limiting factor of all the tested SSM-MFCs (Fig. 3b,c), they are more illustrative of the limitations displayed by the 1 cm tall electrode. Results show that the anodes of the A conditions enter diffusion limitations around −250 mV vs Ag/AgCl, whereas the B and C conditions have a linear slope not encountering limitation problems (Fig. 4e,f). SSM-MFCs mounted with 1 cm tall electrode showed the lower power densities among the bioreactors investigated (Figs. 2 and 4). The anode was significantly hindered and this might be explained by the hypothesis that oxygen, or another oxidant, could interfere with the anode functioning as illustrated by the slightly higher anode OCP. In fact, in the smaller SSM-MFCs, the distance between the anode electrode and the interface air/electrolyte is the smallest among the MFCs investigated. Therefore, a probable negative interaction between oxygen or another oxidant and the anodic anaerobic environment is likely to have been occurring. Also the cathode electrode separately had lower performance compared to B and C conditions. This might be due to a faster biofilm colonisation of the cathode due to its smaller size and this might affect the oxygen accessibility to the cathode catalytic sites therefore lowering the electrode kinetics. These cathode electrodes operate in harsh environments and therefore smaller electrodes are subject to faster organic/inorganic fouling that lower the performance in relatively short amounts of time. As SSM-MFC in this study were operating with human urine, it might be possible that precipitation of struvite or other inorganic salts were occurring on the electrode and thereby limiting the catalytic sites on the cathode. Inorganic and biological fouling has been shown to affect negatively the performance of the cathode [26,49].

**Fig. 5:**
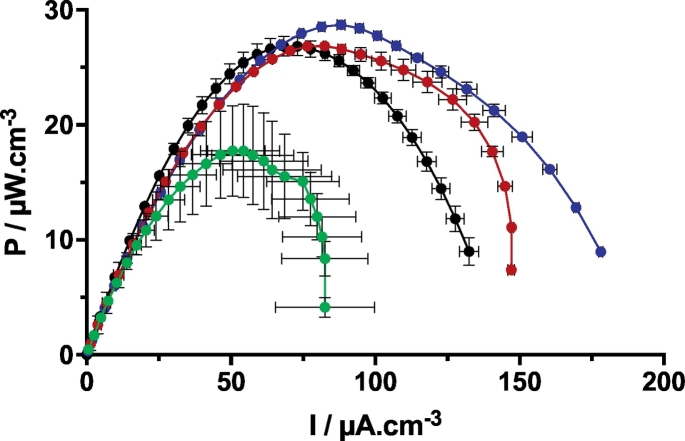
Comparison of the normalised power curves of the SSM-MFCs tested in this study (1 cm, 2 cm and cm conditions; Fig. 4a) and larger ones tested in a previous study (i.e. 4.5 cm tall electrodes [32]). The values have been normalised by the total volume of electrolyte. Green, red and blue curves stand for the 1 cm, 2 cm and 3 cm conditions, respectively conditions A, B and C. Data related to 4.5 cm (black curve) were adapted from [32] CC BY 4.0 (https://creativecommons. org/licenses/by/4.0/). (For interpretation of the references to colour in this figure legend, the reader is referred to the web version of this article.)

The results presented here imply that the scale down of the bioreactor reached a threshold below which the power density decreased. This threshold was identified to be between 1 cm and 2 cm tall electrodes in the present system. Nevertheless, these results confirm that SSM-MFCs can be scaled, up or down depending on implementation needs, without power density losses up to 2 cm tall electrodes. The volumetric power density of the SSM-MFCs tested here could also be compared to the duplicate SSM-MFCs tested in a previous study (i.e. 4.5 cm tall cathodes; ¾ immersion height of the cathodes; 130 ml of electrolyte; [32]) and further supports the hypothesis that SSM-MFCs are scalable in height with no power density losses (Fig. 5). It may be argued that the optimum value for this parameter would be between B and C conditions. However, as the SSM-MFCs of this study and the ones of the previous study were not performed under identical conditions (e.g. HRT), the slight differences between their maximum power transfer point do not reflect the sole change in the electrode height. The values however, fall in the same range, thus confirming the scalability of the system.

## 4 Conclusions

The implementation of microbial fuel cell technology as a urine treatment process requires the technology to be scaled up or down in all dimensions to allow optimum functionality in the target application. In the present work, SSM-MFCs mounted with carbon veil anode and activated carbon cathode were investigated. The aim of this work was to demonstrate that SSM-MFCs could also be scaled in height without power density losses. Among the three tested conditions, the B and C conditions had similar normalised electrochemical performance. Conversely, the SSM-MFCs mounted with 1 cm tall electrodes showed lower densities. However, the measured variations for the 1 cm conditions (i.e. 834 ± 191 μW) indicates that this electrode height is close to the threshold at which the redox potential stratification of the urine column does not match the physical structure of the system. Hence, the performance of this type of MFC is probably hindered at this scale due to the lack of physical separation (i.e. membrane). A further study at the millimetre scale, within the 1 cm experimental condition (A conditions) would enable a more in-depth investigation in order to better define this limit, which is specific to self-stratifying systems; this will be part of our future line of experiments. The most probable reason, as shown by higher anodic potentials and lower anodic kinetics of the 1 cm condition, is the interference with the anode of the oxygen diffusing from the atmosphere, at the top of the urine column. With regard to the pre-treatment of urine to reduce the energy demand of a wastewater treatment plant, the results presented here clearly show that SSM-MFCs can be scaled in height, without power density losses. However, the lower height threshold to which SSM-MFCs can be scaled-down to without power density losses is between the 1 cm and the 2 cm conditions.

## Acknowledgements

The authors would like to acknowledge the 10.13039/100000865Bill & Melinda Gates Foundation for funding the scientific work (grant no. OPP1149065). The authors also thank Patrick Brinson for building the electronic circuitry allowing the microbial fuel cells to be kept under constant voltage.
